# Childhood-Onset Myoclonus-Dystonia Due to *KCTD17* Mutation: A Case Report and Review of Diagnostic Challenges

**DOI:** 10.5334/tohm.1170

**Published:** 2026-04-09

**Authors:** Yun Lin, Yu Aoh, Ming-Kuei Lu

**Affiliations:** 1 Division of Parkinson’s Disease and Movement Disorders, Department of Neurology, China Medical University Hospital, Taichung, Taiwan; 2 Graduate Institute of Biomedical Sciences, College of Medicine, China Medical University, Taichung, Taiwan; 3 Neuroscience and Brain Disease Center, China Medical University, Taichung, Taiwan; 4 School of Medicine, College of Medicine, China Medical University, Taichung, Taiwan; 5 Division of Neurology, Taichung Municipal Geriatric Rehabilitation General Hospital, China Medical University, Taichung, Taiwan

**Keywords:** Myoclonus-dystonia, dystonia, myoclonus, movement disorders, KCTD17 protein, child

## Abstract

**Background:**

Myoclonus–dystonia (M-D) is a rare hyperkinetic movement disorder most commonly associated with *SGCE* mutations, while *KCTD17* represents a less frequent but distinct genetic cause.

**Case Report:**

A 23-year-old man with childhood-onset Tourette-like symptoms developed progressive dystonia, upper-limb–predominant dystonia, upper-limb–predominant myoclonus, and laryngeal involvement. Genetic testing identified a pathogenic *KCTD17* mutation in the patient and his affected mother.

**Discussion:**

This case illustrates that Tourette-like manifestations may occur as an early presentation of *KCTD17*-related myoclonus–dystonia and underscores the importance of longitudinal reassessment and genetic evaluation.

**Highlights:**

This case describes a genetically confirmed *KCTD17*-related myoclonus–dystonia presenting with early Tourette-like features. It highlights the diagnostic complexity arising from overlapping phenomenology, the importance of longitudinal reassessment, and the role of genetic testing in atypical, progressive, or treatment-refractory tic-like presentations.

## Introduction

Myoclonus–dystonia (M-D) is a rare hyperkinetic movement disorder characterized by the combination of non-epileptic myoclonic jerks and dystonia, with onset most commonly in childhood or adolescence. In most patients, symptoms predominantly involve the upper body, including the neck and arms. Although the majority of genetically confirmed cases are associated with mutations in *SGCE*, pathogenic variants in *KCTD17* have increasingly been recognized as an important and distinct cause of autosomal dominant myoclonus–dystonia [2,3,4,5,6].

Differentiating M-D from other childhood-onset hyperkinetic disorders can be challenging, particularly when vocalizations and partially suppressible movements are present. Such features may resemble primary tic disorders and result in diagnostic delay. While tics are typically non-progressive and frequently associated with premonitory urges, genetically determined myoclonus–dystonia is characteristically progressive and may evolve over time with increasing dystonic prominence. Progressive dystonia, positive family history, absence of a premonitory urge, and lack of rebound after suppression may aid diagnosis.

Here, we report a genetically confirmed case of childhood-onset *KCTD17*-related myoclonus–dystonia that initially presented with Tourette-like features and was diagnosed as Tourette’s syndrome in early childhood. The subsequent phenotypic evolution and genetic confirmation highlight the diagnostic challenges posed by overlapping phenomenology and underscore the importance of longitudinal reassessment and targeted genetic testing in patients with atypical, progressive, or treatment-refractory tic-like presentations.

## Case Description

A 23-year-old man was referred to our movement disorders clinic for evaluation of long-standing involuntary movements that began in childhood and had gradually progressed. He was born with a ventricular septal defect that required no intervention. Early neurodevelopmental milestones were reportedly normal.

At 8 years of age, he developed intermittent, abrupt, jerky movements predominantly involving the upper limbs, accompanied by facial grimacing and brief, involuntary vocalizations. The vocalizations and some movements could be partially suppressed with focused effort; however, suppression was incomplete and not followed by rebound once attention was released. He also exhibited repetitive, seemingly purposeless motor behaviors, including shoulder shrugging, head tilting, chest hitting, and kicking surrounding objects. Symptoms were intermittent and fluctuated in severity.

He was evaluated by a pediatrician and diagnosed with Tourette’s syndrome. Treatment with aripiprazole and risperidone resulted in partial reduction of vocalizations but did not halt progression of the abnormal movements. Over subsequent years, the involuntary movements became increasingly persistent. During exacerbations, he experienced falls due to sudden jerks. Hand myoclonus interfered with fine motor tasks, and abrupt jerks would cause him to lose control of objects such as pencils during writing.

Functional impairment became more evident during adolescence. He attended school irregularly due to impaired mobility and frequent symptom-related disruptions. His academic performance declined and remained below average. By 18 years of age, he experienced generalized limb torsion and sustained muscle contractions on most days. Involuntary contractions of the masticatory muscles interfered with chewing, at times necessitating a soft diet. His voice became tense and strained, prompting referral to our clinic for further evaluation.

He denied alcohol consumption, and therefore responsiveness to alcohol could not be assessed. He reported no history of exposure to dopamine-blocking agents prior to symptom onset, no perinatal complications, and no history suggestive of encephalitis or toxin exposure.

Family history was notable for an older brother with intellectual disability and generalized involuntary movements with sustained muscle contractions and twisted postures since childhood. His mother had schizophrenia with visual hallucinations and persecutory delusions and was observed to have intermittent upper-limb jerks on inspection, of which she was unaware (Video 1). This family history suggested autosomal dominant inheritance.

**Video 1:** The patient’s mother demonstrates intermittent upper-limb jerks of which she is unaware. Mild bradykinesia and dysdiadochokinesia are more prominent in the left upper limb. She is currently receiving antipsychotic medication for the management of schizophrenia.

Neurological examination revealed intact cognition and preserved cortical function. Cranial nerve examination, muscle strength, deep tendon reflexes, sensation, and coordination were unremarkable. The predominant findings were generalized dystonia with intermittent vocalizations, upper limb–predominant myoclonus and chorea, and occasional tremulous movements affecting both hands (Video 2). There was no bradykinesia, and the pull test was normal.

**Video 2:** Generalized dystonia with intermittent vocalizations and upper limb–predominant myoclonic jerks before treatment, followed by notable improvement after administration of amantadine and clonazepam.

Laboratory investigations, including metabolic, endocrine, and autoimmune studies, were unremarkable, with no evidence of Wilson’s disease. Brain magnetic resonance imaging was normal.

Given the positive family history suggestive of hereditary dystonia–myoclonus, whole-exome sequencing was performed and did not identify pathogenic variants commonly associated with hereditary dystonia, including *TOR1A, GCH1, SGCE*, and *ANO3*. Subsequent targeted Sanger sequencing identified a heterozygous c.413G>A (p.Arg138His) mutation in the *KCTD17* gene, which has been classified as pathogenic according to ACMG criteria and previously reported in association with myoclonus–dystonia. Both the patient and his mother were heterozygous for this variant, supporting maternal inheritance.

Treatment with amantadine and clonazepam resulted in partial improvement of myoclonus and dystonia. Despite residual physical limitations, he completed regular schooling, adapted socially, and ultimately obtained stable employment as a software engineer.

## Discussion

This case underscores the diagnostic challenge of early Tourette-like manifestations evolving into genetically defined myoclonus–dystonia.

Tourette’s syndrome is characterized by multiple motor tics and at least one vocal tic with onset before 18 years of age. Tics are typically brief, stereotyped, non-progressive, and suppressible, and are often associated with premonitory urges and rebound phenomena [1]. In contrast, myoclonus–dystonia is characterized by non-epileptic myoclonic jerks and dystonia that tend to progress over time and are not associated with typical tic phenomenology. The developmental time course therefore provides an important diagnostic clue: whereas primary tic disorders tend to fluctuate and often improve over time, myoclonus–dystonia is typically progressive, with dystonia becoming more prominent with age. In our patient, the subsequent emergence of sustained generalized dystonia and progressive functional impairment was clinically more consistent with myoclonus–dystonia. Both conditions may present with childhood onset and jerky or tic-like movements predominantly affecting the upper body. Although psychiatric comorbidities have been reported in both disorders, our patient did not exhibit prominent obsessive-compulsive or other psychiatric features.

While phonic tics are a diagnostic hallmark of Tourette syndrome, abnormal vocalizations may also occur in myoclonus–dystonia, particularly in the presence of laryngeal myoclonus or mixed vocal dystonia. A single reported case of *SGCE*-related myoclonus–dystonia has been described as initially misdiagnosed as Tourette syndrome, in which throat clearing was ultimately attributed to laryngeal myoclonus [9].

In addition to involuntary vocalizations, our patient exhibited repetitive motor behaviors during childhood, including chest hitting, shoulder shrugging, head tilting, and kicking surrounding objects. The abrupt and non-patterned quality of these movements, without clear goal-directedness, raises the possibility that they represented upper-limb myoclonic jerks. However, the reported partial suppressibility of the behaviors may be more characteristic of tic phenomenology. Furthermore, patients with Tourette syndrome frequently describe a premonitory urge preceding motor tics, which was absent in our patient. These early features were tic-like but not fully consistent with classical Tourette syndrome.

The differential diagnosis of combined dystonia and myoclonus is broad and includes *SGCE*-related myoclonus–dystonia, *KCTD17*-related myoclonus–dystonia, *ANO3*-related dystonia, *GCH1*-related (dopamine-responsive) dystonia, and secondary causes such as Wilson’s disease, neuroacanthocytosis, and neurodegeneration with brain iron accumulation [2]. In this patient, the absence of parkinsonian features, lack of diurnal fluctuation, normal neuroimaging, negative metabolic workup, and prior genetic testing excluding common hereditary dystonia genes made these alternative diagnoses less likely.

Among genetic causes of myoclonus–dystonia, *SGCE*-related and *KCTD17*-related disorders share overlapping clinical features but demonstrate several distinctions. *SGCE*-related myoclonus–dystonia typically presents before the age of 10 years with proximal upper-limb myoclonic jerks and focal dystonias, and is frequently associated with psychiatric comorbidities [3,4]. In contrast, *KCTD17*-related myoclonus–dystonia usually presents in adolescence or early adulthood, with a more generalized distribution that may include the lower limbs and larynx, fewer psychiatric manifestations, and a less consistent response to alcohol [3,6]. In reported *KCTD17*-mutated cases, myoclonus may be milder or less generalized compared to those with *SGCE* variants [3,5]. The clinical features observed in our patient—including progressive generalized dystonia, laryngeal involvement, upper-limb–predominant myoclonus, and autosomal dominant inheritance—were therefore considered more consistent with *KCTD17*-related myoclonus–dystonia than with classical *SGCE*-related disease. Genetic testing ultimately identified a heterozygous *KCTD17* variant and established the diagnosis, thereby resolving the long-standing diagnostic uncertainty [3]. Whereas myoclonus is the initial symptom in most reported *KCTD17*-related cases, tremulous movements are often described as “jerky” [3]. Isolated tremor has not been specifically reported in the literature, to our knowledge. Although dyskinetic movements have been described in association with dystonia, chorea has not been reported as a presenting manifestation in *KCTD17*-related disorders.

The identified heterozygous *KCTD17* variant c.413G>A (p.Arg138His) has been previously reported in a number of affected individuals with autosomal dominant myoclonus–dystonia, corresponding to the p.Arg145His mutation originally described by Mencacci et al [3]. The difference in amino acid numbering reflects transcript version updates. The variant is absent from large population databases including gnomAD (v4.1.0), supporting rarity (PM2). Multiple in silico prediction tools indicate a deleterious effect on protein function, including a high CADD score (33), ClinPred score of 0.994, and AlphaMissense score of 0.984 (PP3). Functional evidence supporting the role of this variant in disease pathogenesis has been previously reported (PS3) [3,7,8]. According to ACMG/AMP guidelines, the variant meets criteria PS3, PM2, and PP3, supporting a likely pathogenic classification.

Recognition of myoclonus–dystonia is essential, as treatment strategies differ from those used for primary tic disorders. When vocalizations arise from laryngeal dystonia as part of a dystonic movement disorder, treatments targeting tic disorders—including behavioral therapy, α2-agonists, and antipsychotics—may have limited efficacy [1]. Early and accurate diagnosis therefore has important implications for treatment selection, genetic counseling, and long-term management.

This patient has genetically confirmed *KCTD17*-related myoclonus–dystonia. However, the possibility of coexisting or preceding Tourette syndrome cannot be fully excluded, particularly given the early presence of partially suppressible motor and vocal phenomena. The early childhood manifestations were clinically compatible with Tourette syndrome, whereas the subsequent disease course was characterized by progressive generalized myoclonus and sustained dystonia consistent with myoclonus–dystonia. At present, there is insufficient evidence to implicate *KCTD17* directly in primary tic disorders. These observations highlight the importance of longitudinal clinical evaluation and consideration of genetic testing in patients with atypical, progressive, or treatment-refractory Tourette-like presentations.
